# Mapping the Distribution of Invasive *Staphylococcus aureus* across Europe

**DOI:** 10.1371/journal.pmed.1000205

**Published:** 2010-01-12

**Authors:** Franklin D. Lowy

**Affiliations:** College of Physicians & Surgeons of Columbia University, New York, New York, United States of America

## Abstract

Franklin Lowy discusses a new study in *PLoS Medicine* in which the investigators developed an interactive tool for analyzing the spatial distribution of invasive *Staphylococcus aureus*.

**Linked Research Article**

This Perspective discusses the following new study published in *PLoS Medicine*:

Grundmann H, Aanensen DM, van den Wijngaard CC, Spratt BG, Harmsen D, et al. (2010) Geographic Distribution of *Staphylococcus aureus* Causing Invasive Infections in Europe: A Molecular-Epidemiological Analysis. PLoS Med 7(1): e1000215. doi:10.1371/journal.pmed.1000215

Understanding the geographic distribution of microbial pathogens such as *Mycobacterium tuberculosis*, *Salmonella* spp., or *Staphylococcus aureus* is critical to developing strategies to reduce their transmission. Varied approaches have been used to track their spread including the use of national surveillance networks, multinational strain collections, or more targeted strategies that track social networks within high-risk populations [1]–[4]. The integration of molecular techniques with these surveillance strategies helps identify specific bacterial strains or clones and potentially provides further insight into strain transmission and virulence.

For *S. aureus*, understanding the transmission pathways of the different clones remains of critical importance. In the past 10 years the number of antibiotic-resistant staphylococci has increased both in the health care setting and in the community [5]–[7]. Despite the availability of effective antimicrobials, invasive *S. aureus* infections continue to cause significant morbidity and mortality. This burden of disease was highlighted in a recent report from the United States that estimated the overall incidence of invasive methicillin-resistant *S. aureus* (MRSA) infections at 31.8 per 100,000 and the overall mortality from these infections at 6.3 per 100,000 [8].

Understanding the epidemiology of these infections, the role of specific strains and the basis for their spread has therefore continued to be an area of intense interest. Earlier studies have shown that, since the first report of a methicillin-resistant staphylococcus in 1961, only a limited number of MRSA clones have been responsible for the bulk of infections worldwide suggesting that these unique clones have a survival advantage [9],[10]. Learning how these clones spread, target selected populations, and cause disease will provide insight into understanding the basis for their remarkable persistence and virulence.

## A New Study on the Spatial Distribution of *S. aureus*

In a new study by Hajo Grundmann and colleagues in this issue of *PLoS Medicine*, the investigators describe the development of an interactive tool for analyzing the spatial distribution of invasive *S. aureus* [11]. The authors assembled a large group of collaborators throughout much of Europe including 450 hospitals from 26 countries. They then standardized strain collection and typing procedures. This arduous start-up process is likely to provide payoffs well beyond this initial study. Over a 6-month period, from 2006–2007, participating hospitals provided five successive methicillin-resistant and methicillin-susceptible *S. aureus* (MSSA) isolates collected from patients with invasive infections. Most of these isolates were from bacteremias. These isolates were characterized using a sequence-based approach, *spa* typing (based on the polymorphic protein A gene that is uniformly found in *S. aureus*), which allows for identification of specific strains. The strain *spa* types were assigned to their original hospital collection site and the spatial analytic tools were then used to map common *spa* types both by their regional proximity and their country of origin.

Not surprisingly the authors found that the MSSA isolates were more varied and more widely dispersed than the MRSA isolates. The latter were more geographically limited and had fewer clonal types. The MRSA *spa* types tended to mostly cluster within regional borders and, in several instances, were associated with individual hospitals, suggesting an outbreak. The authors provide an interactive map that allows others to query the web site for the location of particular clones and to examine the distribution throughout Europe of the different strains (http://www.spatialepidemiology.net/SRL-Maps).

The study has some limitations, many already noted by the authors. There was a potential selection bias given the reduced number of MRSA versus MSSA isolates. In an effort to identify specific strains with enhanced virulence, the authors linked strain profiles with all-cause mortality at 2 weeks. While no association was found, all-cause mortality is a relatively crude indicator and may miss factors contributing to virulence that are not reflected in death as an outcome. Future studies will likely further refine this approach. Missing data, especially on the source of the isolates (community versus hospital), and the all-cause mortality may have influenced the analysis of this data.

## What Is the Potential of This Approach?

The major strength of Grundmann and colleague's proof of principle study is that it shows the potential value of this integrated approach. By establishing a large collaborative network and then combining molecular and spatial analytic techniques, the authors illustrate the ability to map specific strains across large geographic regions. At its most basic, the study provides information on whether clones of *S. aureus* are randomly distributed or clustered. The authors however also illustrate that there are many other potential applications of this approach.

The authors show that by linking strain profiles with patient outcomes they can generate information on the potential virulence of a particular clone. In the future, this approach could be applied to strains isolated from the community, strains from different tissue sites, or to strains with or without selected virulence genes. Sociodemographic information, such as the age, sex, or occupation of the patient, can be correlated with the strain type. This information may then generate hypotheses on transmission pathways, susceptible high-risk occupations (e.g., pig farmers and *spa* type t034 ), or particularly vulnerable populations [12],[13].

Innovative mapping strategies, based on information provided from international organizations, news media, and scientific reports, have recently been used to develop interactive maps that show the geographic distribution of a variety of different pathogens (e.g., see Health Map, http://www.healthmap.org/en). Grundmann and colleagues' study is one of several recent investigations that illustrate the ability of spatial mapping techniques to help understand the spread of new or reemerging pathogens at the local as well as the international level [14],[15].

## Abbreviations

MRSA: methicillin-resistant *S. aureus*
MSSA: methicillin-susceptible *S. aureus*

## References

[1] Schuchat A, Hilger T, Zell E, Farley MM, Reingold A (2001). Active bacterial core surveillance of the emerging infections program network.. Emerg Infect Dis.

[2] Klovdahl AS, Graviss EA, Yaganehdoost A, Ross MW, Wanger A (2001). Networks and tuberculosis: an undetected community outbreak involving public places.. Soc Sci Med.

[3] Friedrich AW, Daniels-Haardt I, Kock R, Verhoeven F, Mellmann A (2008). EUREGIO MRSA-net Twente/Munsterland–a Dutch-German cross-border network for the prevention and control of infections caused by methicillin-resistant *Staphylococcus aureus*.. Euro Surveill.

[4] Fluit AC, Wielders CL, Verhoef J, Schmitz FJ (2001). Epidemiology and susceptibility of 3,051 *Staphylococcus aureus* isolates from 25 university hospitals participating in the European SENTRY study.. J Clin Microbiol.

[5] Chambers HF (2001). The changing epidemiology of *Staphylococcus aureus*?. Emerg Infect Dis.

[6] Kaplan SL, Hulten KG, Gonzalez BE, Hammerman WA, Lamberth L (2005). Three-year surveillance of community-acquired *Staphylococcus aureus* infections in children.. Clin Infect Dis.

[7] Klevens RM, Edwards JR, Tenover FC, McDonald LC, Horan T (2006). Changes in the epidemiology of methicillin-resistant *Staphylococcus aureus* in intensive care units in US hospitals, 1992–2003.. Clin Infect Dis.

[8] Klevens RM, Morrison MA, Nadle J, Petit S, Gershman K (2007). Invasive methicillin-resistant *Staphylococcus aureus* infections in the United States.. JAMA.

[9] Jevons MP (1961). “Celbenin”-resistant staphylococci.. BMJ.

[10] Oliveira DC, Tomasz A, de Lencastre H (2002). Secrets of success of a human pathogen: molecular evolution of pandemic clones of meticillin-resistant *Staphylococcus aureus*.. Lancet Infect Dis.

[11] Grundmann H, Aanensen DM, van den Wijngaard CC, Spratt BG, Harmsen D Geographic distribution of *Staphylococcus aureus* causing invasive infections in Europe.. PLoS Med.

[12] Voss A, Loeffen F, Bakker J, Klaassen C, Wulf M (2005). Methicillin-resistant *Staphylococcus aureus* in pig farming.. Emerg Infect Dis.

[13] Mishaan AM, Mason EO, Martinez-Aguilar G, Hammerman W, Propst JJ (2005). Emergence of a predominant clone of community-acquired *Staphylococcus aureus* among children in Houston, Texas.. Pediatr Infect Dis J.

[14] Wylie JL, Cabral T, Jolly AM (2005). Identification of networks of sexually transmitted infection: a molecular, geographic, and social network analysis.. J Infect Dis.

[15] Tirabassi MV, Wadie G, Moriarty KP, Garb J, Konefal SH (2005). Geographic information system localization of community-acquired MRSA soft tissue abscesses.. J Pediatr Surg.

